# Sibling Species Composition and Susceptibility Status of *Anopheles gambiae* s.l. to Insecticides Used for Indoor Residual Spraying in Eastern Uganda

**DOI:** 10.1155/2023/2225233

**Published:** 2023-07-10

**Authors:** Julius Iga, Stephen Ochaya, Richard Echodu, Elizabeth A. Opiyo, Alex K. Musiime, Angella Nakamaanya, Geoffrey M. Malinga

**Affiliations:** ^1^Department of Biology, Faculty of Science, Gulu University, P.O. Box 166 Gulu, Uganda; ^2^Department of Immunology and Microbiology, Faculty of Medicine, Gulu University, P.O. Box 166 Gulu, Uganda; ^3^National Malaria Control Division, Ministry of Health, Uganda; ^4^Uganda Virus Research Institute, P.O. Box 49 Entebbe, Uganda

## Abstract

**Background:**

Malaria remains one of the most critical disease causing morbidity and mortality in Uganda. Indoor residual spraying (IRS) and the use of insecticide-treated bed nets are currently the predominant malaria vector control interventions. However, the emergence and spread of insecticide resistance among malaria vectors threaten the continued effectiveness of these interventions to control the disease, particularly in high transmission areas. To inform decisions on vector control, the current study evaluated the *Anopheles* malaria vector species and their susceptibility levels to 0.1% bendiocarb and 0.25% pirimiphos-methyl insecticides used in IRS intervention program in Namutumba district, Eastern Uganda.

**Methods:**

*Anopheles* larvae were collected between March and May 2017 from different breeding sites in the parishes of Nsinze and Nawaikona in Nsinze sub-county and reared to adults to assess the susceptibility status of populations in the study area. Mosquitoes were identified using morphological keys and species-specific polymerase chain reaction (PCR) assays. Susceptibility tests were conducted on 2- to 5-day-old non-blood-fed adult female *Anopheles* that emerged using insecticide-impregnated papers with 0.1% bendiocarb and 0.25% pirimiphos-methyl following standard World Health Organization (WHO) insecticide susceptibility bioassays. A Log-probit regression model was used to derive the knock-down rates for 50% and 95% of exposed mosquitoes.

**Results:**

A total of 700 mosquito larvae were collected from different breeding sites. Morphological identification showed that 500 individuals that emerged belonged to *Anopheles gambiae* sensu lato (s.l.), the main malaria vector. The PCR results showed that the dominant sibling species under the *A. gambiae* complex was *Anopheles arabiensis* 99.5% (395/397). WHO bioassay tests revealed that the population of mosquitoes exhibited high levels of susceptibility (24-hour post-exposure mortality 98–100%) to both insecticides tested. The median knock-down time, KDT_50_, ranged from 6.6 to 81.4 minutes, while the KDT_95_ ranged from 21.6 to 118.9 minutes for 0.25% pirimiphos-methyl. The KDT_50_ for 0.1% bendiocarb ranged from 2.8 to 62.9 minutes, whereas the KDT_95_ ranged from 36.0 to 88.5 minutes.

**Conclusions:**

These findings indicate that bendiocarb and pirimiphos-methyl are still effective against the major malaria vector, *A. arabiensis* in Nsinze sub-county, Namutumba district, Uganda and can be effectively used for IRS. The study has provided baseline information on the insecticide susceptibility status on malaria vectors in the study area. However, routine continuous monitoring program of insecticide susceptibility and malaria vector composition is required so as to guide future decisions on insecticide use for IRS intervention toward malaria elimination and to track future changes in vector population.

## 1. Background

Malaria remains one of the most important disease-causing morbidity and mortality in Uganda [1–3]. Uganda has the third highest number of malaria cases recorded annually in Sub-Saharan Africa [4] as well as some of the highest reported malaria transmission rates in the world, with approximately 16 million reported cases in 2013 [3], and over 10,500 estimated deaths annually [5]. Among the numerous *Anopheles* species present in the country, *Anopheles gambiae* s.l. and *Anopheles funestus* s.l. constitute the principal malaria vectors [5, 6] with *A. gambiae* s.l. being the main vector species in most parts of the country [6–8].

Pyrethroid-treated long-lasting insecticide-treated nets (LLINs) and indoor residual spraying (IRS) are currently the key malaria vector control measures and elimination efforts in Uganda [9–11]. However, these measures are hampered by the rapid emergence and geographical spread of insecticide resistance to recommended classes of insecticides [3]. Planning a large-scale programme of vector control requires a more detailed knowledge on the composition of the vector species and their susceptibility to available insecticides [3]. Studies from Kenya [12, 13], Tanzania [14], and Equatorial Guinea [15] have indicated a shift from *A. gambiae* s.s. and *A. funestus* malaria vectors to *Anopheles arabiensis* following IRS and continuous usage of LLINs. In Uganda, a shift from *A. gambiae* s.s. to *A. arabiensis* in Tororo [16] and other parts of the country [17, 18] has also been reported.

Resistance to pyrethroid insecticides used for adult malaria vector control has been reported in several parts of Uganda [18–23] and from other African countries including Kenya [22, 24] and Tanzania [25]. Resistance to 0.25% bendiocarb has also been detected in some areas of Soroti district [19]. The high rate at which mosquitoes and parasites develop resistance to insecticides and anti-malarial medicines [26] is likely to frustrate and stall efforts in the fight against malaria. Unfortunately, data on insecticide susceptibility in Uganda and the malaria vector composition, particularly in areas where IRS is implemented, are still limited. Accurate and routine monitoring of the susceptibility status of major malaria vectors to recommended insecticide is critical to inform control strategies and to evaluate the effectiveness of the current management interventions. The current study aimed to identify *A. gambiae* sibling species responsible for malaria transmission in Nsinze sub-county using molecular tools and to establish their susceptibility to 0.1% bendiocarb and 0.25% pirimiphos-methyl, which are among the World Health Organization (WHO) insecticides recommended for IRS. The findings of this study will serve as a baseline for guiding future policy and decision-making on public health insecticide use for IRS in the country.

## 2. Methods

### 2.1. Study Area

**Figure 1:**
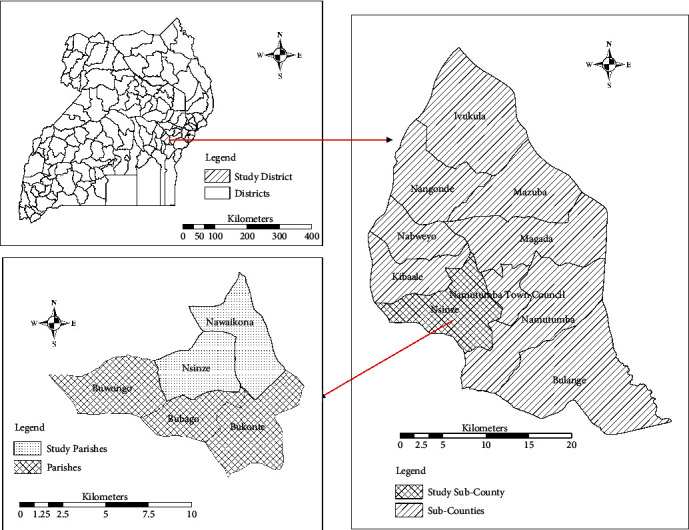
Location of sites used for larval collection at Namutumba district, Uganda in 2017.

The study was conducted in the parishes of Nsinze and Nawaikona, Nsinze sub-county, Namutumba district, Eastern Uganda (latitude 0°52′N and longitude 33°40′E, 814.3 km^2^, (Figure 1). These parishes are situated at a distance of 13.6 km from each other, in an area of intensive subsistence crop production and animal husbandry with rice, maize, millet, and sweet potatoes as the main crops. The areas have numerous temporary and semi-permanent mosquito breeding sites. They are among the malaria high transmission districts in the country, with IRS and LLIN distribution employed as the main malaria control interventions.

### 2.2. Mosquito Sampling and Larval Rearing

Potential breeding habitats (temporary stagnant rain water pools, semi-permanent waters, fresh, sunlit, shallow waters, and temporary pools in rice fields) in the two parishes were inspected for the presence of mosquito larvae. The positive habitats were sampled using a WHO standard mosquito dipper (11.5 cm diameter and 350 ml capacity) [27]. Sampling took place between March and May 2017, during the rainy season. Dry season sampling was not conducted due to limited funds. The larvae collected from various breeding sites in the two parishes were reared separately to adults in the field insectary with temperatures between 23.3°C and 27°C and relative humidity between 54% and 92%. The larvae were not fed on any diet other than them feeding on the nutrients in the water from the breeding sites where they were collected from. The emerging adult female *A. gambiae* s.l. was morphologically identified using standard taxonomic keys developed by Coetzee [28]. They were later sorted, culex mosquitoes removed, and the *Anopheles* fed on 10% glucose syrup solution, and 2–5 days old female mosquitoes were used for insecticide susceptibility tests.

### 2.3. Insecticide Susceptibility Bioassay

The susceptibility of *A. gambiae* s.l. mosquitoes to diagnostic concentrations of 0.1% bendiocarb and 0.25% pirimiphos-methyl was performed according to the standardized WHO protocol [26, 29], at temperatures between 23.3°C and 27°C and relative humidity between 54% and 92%. For each study site, 20 individuals of 2 to 5 days old non-blood-fed adult females *A. gambiae* s.l. in five replicates were aspirated into holding tubes and exposed to insecticide-impregnated test papers with discriminating doses of bendiocarb (0.1%) and pirimiphos-methyl (0.25%) and there was one control with equal number of mosquitoes exposed to papers impregnated with olive oil and acetone without insecticide, respectively. These insecticides were selected based on their current operational importance in the IRS national malaria control program. Pirimiphos-methyl and bendiocarb are currently the insecticides used for IRS in Eastern Uganda [30]. The number of knock-down mosquitoes was recorded for each insecticide over the 1-hour exposure period at 10, 15, 20, 30, 40, 50, and 60 minutes [29]. After 60 minutes of exposure, all mosquitoes were transferred back into holding tubes and fed on 10% glucose solution in soaked cotton pads. The proportions of dead and surviving mosquitoes (final mortality) were recorded after a 24-hour post-exposure holding period. After the bioassays, all mosquitoes, both dead and alive, were individually packed in 1.5-ml Eppendorf tubes and preserved in silica gel for further molecular analyses using polymerase chain reaction (PCR).

#### 2.3.1. Identification and Molecular Characterization of Female *Anopheles* Mosquitoes

All adult mosquitoes were morphologically identified to species level using standard taxonomic identification keys [28] under a high-powered dissecting microscope. Features on wings, palps, abdomen, and legs were used for identification. Morphologically identified *A. gambiae* s.l. adult female mosquito samples (both dead and surviving) tested for susceptibility to insecticides were identified as sibling species using species-specific PCR assay following the methods of Scott et al. [31]. The analyses were done at the Molecular Biology Laboratory, Makerere University, Uganda. Susceptible *A. arabiensis* and *A. gambiae* s.s. strain obtained from Biodefense and Emerging Infection Research Resources Repository resources were used as a positive control, and a tube without mosquito leg but containing all reagents used in making the master mix was used as a negative control. PCR amplification of DNA from legs of 397 female *Anopheles* mosquitoes after exposure to insecticide-treated papers was performed as previously described [26]. Primers specific to *A. gambiae* s.s. (CTGGTTTGGTCGGCACGTTT), *A. arabiensis* (AAGTGTCCTTCTCCATCCTA), and universal *A. gambiae* s.l. (GTGTGCCCCTTCCTCGATGT) complex were used in this study. Amplification of single mosquito leg with master mix consisting of 1 unit of GoTaq, Green Tag buffer (Promega, Madison, MO) and primers making a total volume of 25 *μ*l was used per PCR reaction and run in a touch screen thermal cycler (SimpliAmp, Applied Biosystems, Life Technologies, Singapore). Denaturation occurred for 5 minutes at 95°C, followed by 30 cycles of 30 seconds at 95°C, 30 seconds at 50°C, and 30 seconds at 72°C and final extension for 10 minutes at 72°C. The quality of PCR products was assessed using 1.5% ethidium bromide-stained agarose gel, and the band size of PCR products for each species was visualized on a Gel Doc Imaging System (UVITEC, Cambridge). The band sizes were evaluated against a 100 bp DNA ladder molecular weight marker (Life Technologies, Rockville, MD) to confirm the expected molecular weight of the amplification products.

#### 2.3.2. Data Analyses

The status of susceptibility of adult mosquitoes to insecticides after 24 hours post-exposure was determined for each insecticide using percentage mortality. Mosquitoes' susceptibility to insecticides was interpreted based on the WHO [29] criteria. As per the criteria, 24-hour mortality of 98–100% indicates susceptibility, mortality of 90–97% indicates the possibility of resistance or suspected resistance that needs to be confirmed, and mortality less than 90% classified as resistant [26, 29]. The average observed mortality was corrected using Abbot's formula [32] when the control mortality was between 5% and 20%. The exposure time (in minutes) required to achieve 50% and 95% knock-down (KDT_50_ and KDT_95_) and their 95% confidence intervals were estimated for each insecticide using log-time probit regression model according to the method of Finney [33] in GENSTAT.

## 3. Results

### 3.1. Mosquito Species Composition

**Figure 2:**
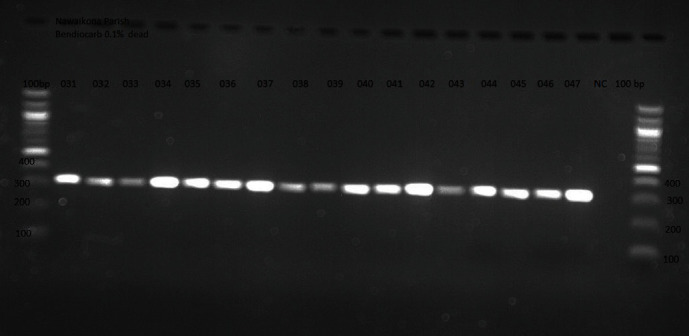
PCR representative gel picture after electrophoresis on 1.5% agarose gel. Lane one is 100 bp: molecular weight ladder, lane 013-047, products of *A. arabiensis* DNA fragments (315 bp), negative control (NC). Note that positive control sample is not displayed on the gel.

A total of 700 mosquito larvae were collected across the two parishes in Nsinze sub-county between March and May 2017. Morphological identification showed that 500 individuals that emerged belonged to *A. gambiae* s.l. PCR analysis on 397 samples showed that *A. arabiensis* was the predominant (395, 99.5% including survivors) malaria vector species (Figure 2). However, two samples failed to amplify.

### 3.2. Insecticide Susceptibility Status of *A. gambiae* s.l. against Different Insecticides

In the present study, batches of 20 mosquitoes in five replicates were exposed in test kits with insecticide-impregnated papers, and a control with equal number of mosquitoes exposed to papers impregnated with silicone oil was run in parallel for 1 hour for each insecticide per parish to determine their susceptibility to insecticides. As per the WHO insecticide susceptibility criterion, the local mosquito populations of *A. gambiae* s.l. in both parishes of Nsinze sub-county were completely susceptible to both 0.25% pirimiphos-methyl and 0.1% bendiocarb (Table 1). The mortality rate of *A. gambiae* s.l. mosquitoes against pirimiphos-methyl in both parishes was 100%, whereas 0.1% bendiocarb exhibited 98% and 100% mortalities in Nsinze and Nawaikona parishes, respectively (Table 1). Mortalities in all the control populations tested were less than 5%, thus no corrections, using the Abbott's formula for mortality rate, were required during data analysis.

### 3.3. Knock-Down Effect

The median knock-down time (KDT_50_) taken for 50% of the test mosquitoes to be knock-down obtained from the time–mortality regression using probit analysis ranged from 2.8 (95% CI: 0.9–4.9) to 6.6 (95% CI: 4.8–8.2) minutes, while the KDT_95_ ranged from 21.6 (95% CI: 18.9–26.1) to 36.0 (95% CI: 28.3–53.9) minutes for 0.1% bendiocarb (Table 2). The longest median KDT_50_ of 6.6 (95% CI: 4.8–8.2) minutes was recorded in Nsinze parish (Table 2). The KDT_50_ for 0.25% pirimiphos-methyl ranged from 62.9 (95% CI: 59.9–67.9) to 81.4 (95% CI: 69.6–189.1) minutes, whereas the KDT_95_ ranged from 88.5 (95% CI: 78.7–109.3) to 118.9 (95% CI: 87.4–660.7) minutes. Like for 0.1% bendiocarb, the longest median KDT_50_ of 81.4 (95% CI: 69.6–189.1) was recorded in Nsinze parish. Considerably, it took a long time for mosquitoes to be knocked down by 0.25% pirimiphos-methyl compared with 0.1% bendiocarb. Nsinze parish generally recorded a higher KDT for all the tested insecticides (Table 2).

## 4. Discussion

The results of this present study revealed the susceptibility of *A. arabiensis* malaria vectors to diagnostic concentrations of 0.1% bendiocarb and 0.25% pirimiphos-methyl used for IRS in Namutumba district, Uganda. Similar findings have been reported elsewhere in the country [5, 18–20, 34]. Hakizimana et al. [35] also reported susceptibility of *A. arabiensis* to bendiocarb in 11 out of the 12 studied sites in Rwanda between 2011 and 2013. Recent studies in Migori county, western Kenya [13] showed *A. arabiensis* to be fully susceptible to pirimiphos-methyl and bendiocarb. Consistent with this study, Matowo et al. [36] also found *A. gambiae* s.l. in Muleba village of Tanzania to be fully susceptible to pirimiphos-methyl. Pirimiphos-methyl was also reported to cause 100% mortality in the most dominant malaria vector, *A. gambiae* s.l. in all studied sites in Uganda [5]. On the contrary, bendiocarb resistance was reported in Soroti, a non-IRS interventional district in Uganda [19]. The high susceptibility of malaria vectors to these insecticides is probably due to the limited use of these insecticides for malaria control in Namutumba, and consequently, mosquitoes are not exposed to them. These results are promising for successful malaria control and justify their (insecticides) continued use in IRS in high transmission districts, including the studied area. This is because of its ability to effectively kill malaria vectors in Uganda, coupled with its longer residual effect [37] on the sprayed wall surfaces (8–9 months, depending on the nature of the sprayed surface).

The results of this study showed that 0.1% bendiocarb had a quicker knock-down effect on exposed mosquitoes than 0.25% pirimiphos-methyl, as observed also in other previous studies in south-eastern Tanzania [38]. Thus, an insecticide such as bendiocarb with a quicker knock-down effect (KDT_50_, 6.6–81.4 minutes) compared to 0.25% pirimiphos-methyl (KDT_50_, 21.6–118.8 minutes) would have a higher preference in malaria vector control programs as it does not allow the mosquito time to survive and transmit malaria.

In the present study, PCR characterization of *A. gambiae* s.l. revealed that the predominant sibling malaria vector species in the study area is *A. arabiensis* (95.5%). Studies in other parts of the country, for example, Tororo [16], Kamuli [39], Jinja [18], have documented sympatric existence of *A. arabiensis* with *A. gambiae* s.s. and *A. funestus* [20]. These three species have also been reported as the main vectors of *Plasmodium falciparum* malaria in Sub-Saharan Africa [8, 25, 40]. However, this is contrary to a study conducted in Nyabushozi county, Western Uganda, where *A. gambiae* s.s. accounted for 93.6% (1544) of the total 2566 *A. gambiae* s.l. examined by PCR, while *A. arabiensis* was absent [7]. Earlier studies conducted in Eastern Uganda [8] suggested that *A. gambiae* s.s. was the predominant species before the scale-up of interventions with LLINs and IRS in 2016. However, recently, a survey conducted in Tororo, Eastern Uganda [19], for example, showed a shift in malaria vector from predominantly *A. gambiae* to *A. arabiensis* after the start of the residual insecticide spraying (IRS) in 2015. Such a shift in species composition has also been reported in Apac District (formerly an IRS zone) [19], and in other African countries, for example, Botswana [41], Kenya [42], and Rwanda [35]. The shift in the composition of *A. gambiae* complex has important implications for the malaria epidemiology and strategies for control of malaria in the study area given that *A. arabiensis* is an opportunistic feeder which tends to rest outdoors but feed on humans and non-human hosts both indoors and outdoors. Therefore, an integrated control of malaria vectors should incorporate both indoor and outdoor interventions, since outdoor/biting mosquitoes have been shown to be less susceptible to indoor interventions such as IRS [35]. Furthermore, the predominance of *A. arabiensis* species in Nsinze sub-county could also be explained by their ability to tolerate a wide range of larval breeding conditions habitats compared to *A. gambiae* s.s. [43, 44]. For example, *A. arabiensis* is known to prefer breeding in temporary and permanent man-made habitats such as rice fields than in other sites [43]. Most of the larvae were collected from running, fresh, sunlit, shallow waters, and temporary pools in rice fields. Their ability to survive in shallow and moving waters [44] could further explain their dominance in this area. The proliferation of *A. arabiensis* larvae in flooded rice fields could be explained by the ability of the vegetation (rice) to act as temporary refugia against strong wave action that could wash away the larvae and also as hiding areas against predators [45]. Finally, the scale-up of IRS, Insecticide-treated bed nets, and other indoor insecticidal public health vector control interventions has been reported to contribute to selection for the more exophilic and zoophagic *A. arabiensis* in most of the interventional areas [5, 16]. From the PCR results, it was observed that two samples did not amplify. This could be that it was another strain unable to be amplified using the current PCR primers, hence the need for sequencing to reveal their identities.

## 5. Conclusions

The findings of this study demonstrate that bendiocarb and pirimiphos-methyl are still effective against the major malaria vector, *A. arabiensis* in Nsinze sub-county, Namutumba district, Uganda and can be effectively used for IRS. The study has provided baseline information on the insecticide susceptibility status on malaria vectors in the study area. However, routine continuous monitoring program of insecticide susceptibility and malaria vector composition is required so as to guide future decisions on insecticide use for IRS intervention toward malaria elimination and to track future changes in vector population.

## Figures and Tables

**Table 1:** WHO insecticide susceptibility bioassay results with *A. gambiae* s.l. populations from Namutumba district, Eastern Uganda between March and May 2017.

Study site	Insecticide	No. exposed	No. dead	No. alive	Percentage mortality∗	Susceptibility status
Nawikona	0.1% Bendiocarb	100	98	2	98	Susceptible
Nsinze	0.1% Bendiocarb	100	100	0	100	Susceptible
Nawikona	0.25% Pirimiphos-methyl	100	100	0	100	Susceptible
Nsinze	0.25% Pirimiphos-methyl	100	100	0	100	Susceptible

∗Mortality recorded 24-hour post-exposure.

**Table 2:** Knock-down times (KDT) and mortality rates of *Anopheles* mosquitoes after exposure to diagnostic concentrations of 0.1% bendiocarb and 0.25% pirimiphos-methyl for 60 minutes.

Study site	Insecticide	Total exposed	No. of replicates	No. dead	KDT_50_ (min) 95% CI	KDT_95_ (%) 95% CI
Nsinze Parish	0.1% Bendiocarb	100	5	98	6.6 (4.8–8.2)	21.6 (18.9–26.1)
Nawaikona Parish	0.1% Bendiocarb	100	5	100	2.8 (0.9–4.9)	36.0 (28.3–53.9)
Nsinze Parish	0.25% Pirimiphos-methyl	100	5	40	81.4 (69.6–189.1)	118.9 (87.4–660.7)
Nawaikona Parish	0.25% Pirimiphos-methyl	100	5	9	62.9 (59.9–67.9)	88.5 (78.7–109.3)

CI, confidence interval; KDT, knock-down time; KDT_50_, time taken for 50% of test mosquitoes to be knocked down; KDT_95_, time taken for 95% of the test mosquitoes to be knocked down.

## Data Availability

The data that support the findings of this study are available from the corresponding author upon request.

## Additional Points

*Study Limitations*. (i) Molecular identification was conducted only on mosquitoes exposed to insecticide-treated papers, and sampling was only from the breeding sites and not households. (ii) The WHO method of susceptibility monitoring employed in this study has the limitation that it is unable to detect low frequency resistance in a population and gives little information on the underlying mechanisms and levels of resistance. (iii) The study was designed and conducted during the rainy season; thus, finding might be different during the dry season. It is recommended for future studies to perform susceptibility bioassays during both wet and dry seasons.
